# Promoting anti-doping behaviors through group norms: understanding the role of team identity among adolescent athletes

**DOI:** 10.3389/fpubh.2026.1788343

**Published:** 2026-02-24

**Authors:** Jiang Du, Xinghua Wang, Shuyue Luo, Xue Xia, Chuansheng Dong

**Affiliations:** 1 Shenyang Sport University, Shenyang, China; 2 Northeastern University, Shenyang, China

**Keywords:** adolescent athletes, anti-doping behaviors, group norms, health education, team identity

## Abstract

**Background:**

While anti-doping education programs have expanded globally, their effectiveness in collectivist cultural contexts remains poorly understood. Existing research has largely focused on individual-level psychological factors, overlooking how group-level social dynamics interact to shape health behaviors in intensive team-based training environments where both normative pressures and identity processes operate simultaneously.

**Objective:**

To examine whether and how group norms influence anti-doping behaviors among Chinese adolescent amateur athletes, and to test team identity as a potential mediating mechanism in this relationship.

**Methods:**

Cross-sectional survey of 1,718 adolescent athletes (mean age 18 years) from sports schools across 15 Chinese cities. Structural equation modeling tested direct effects of group norms on anti-doping behaviors and indirect effects through team identity as a mediator.

**Results:**

Group norms were strongly associated with anti-doping behaviors (*β* = 0.54, *p* < 0.001). However, team identity exhibited a negative mediating pattern in this association (indirect effect = −0.194, accounting for 35.8% of total effect), revealing a paradox: while anti-doping norms show a positive association with healthy behaviors, the concurrent strengthening of team identity is statistically associated with an attenuation of this relationship. This negative mediation suggests that team-level interest coalitions can create conflicting pressures that complicate health promotion efforts in sports settings.

**Conclusion:**

This study identifies a dual-pathway social mechanism through which group norms and team identity jointly shape adolescent athletes’ anti-doping behaviors in collectivist sports settings. From a practical perspective, the findings suggest that anti-doping education programs should move beyond individual-focused knowledge delivery and explicitly incorporate team-level strategies, such as shaping shared norms, engaging peer leaders, and aligning team identity with health and ethical values. Interventions that strengthen team cohesion without addressing underlying performance-driven identity pressures may be insufficient or counterproductive. These findings provide actionable guidance for designing culturally adapted anti-doping education programs that leverage group norms while managing the potential risks associated with strong team identification.

## 1 Background

Protecting adolescent athletes from doping represents a critical public health and health promotion priority. Beyond threatening competitive fairness, doping has significant impacts on athletes’ physical health and psychological state. Traditionally, sports governing bodies and researchers have invested substantial resources in curbing doping through punitive strategies such as limiting access to banned substances and enhancing testing procedures. However, existing research suggests that relying solely on punitive measures is not effective in reducing doping incidence or promoting health behaviors among athletes. Data from the World Anti-Doping Agency (WADA) indicate that global positive doping test results have changed little over the past decade. Martinelli et al. (1) point out that punitive strategies rely primarily on athletes’ fear and anxiety of consequences, which not only exacerbates psychological burdens but may also weaken the construction of a healthy sport culture. These limitations underscore the urgent need for anti-doping efforts to shift from punishment to prevention, with a focus on exploring the psychological and social factors behind athletes’ behaviors to develop more effective health education and promotion interventions. This paradigm shift aligns with global health priorities, including the United Nations Sustainable Development Goals (SDGs), particularly SDG 3 (Good Health and Well-being) and SDG 4 (Quality Education), which emphasize comprehensive health education for young people.

In China, collectivist culture provides a unique context for understanding and promoting health behaviors among young athletes. Amateur sports schools serve as the primary training system for competitive sports reserves and represent distinctive settings for health education interventions. These institutions are centered on team-based training and have shaped strong collective values and group consciousness among athletes. According to statistics, 61% of outstanding athletes and Olympic gold medalists come from amateur sports schools, making this training system an important foundation for competitive sports development in China. However, the long-standing development strategy oriented towards “Olympic glory” has sometimes led athletes, driven by collective interests, to adopt health-compromising behaviors in pursuit of short-term results. Group incidents of doping violations have occurred in some amateur sports schools, exposing critical gaps in anti-doping health education within this youth population and highlighting the need for culturally-adapted health promotion interventions that account for the collectivist context of Chinese sports schools.

The adolescent stage represents a critical developmental window for health education and behavior change interventions. This period is characterized by the formation of values and behavioral norms, as well as heightened sensitivity to group norms and social influences. Research has shown that external environment and social culture have significant impacts on adolescent athletes’ health behaviors. For example, Laure and Binsinger (2) showed that only 1.3–3.0% of adolescent athletes “intentionally dope” and that most adolescents remain “clean” under normal circumstances. However, external environmental factors may be important drivers of the shift from health-promoting to health-compromising behaviors in this population. Erickson et al. (3) found that the psychological impact of learning about peers’ doping use is complex and may lead to increased emotional acceptance of doping behavior, illustrating how social contexts influence health-related decision-making among youth athletes. In China’s cultural context that emphasizes collective values, group norms and team environments may play an even more significant role in shaping adolescent athletes’ health behaviors. This understanding has important implications for designing effective peer-based health education interventions and developing health literacy among young athletes in collectivist settings.

From a health education and promotion perspective, understanding the social-psychological mechanisms that influence health behaviors is essential for developing effective interventions. Existing research indicates that group norms, defined as shared behavioral guidelines within a group, play a critical role in regulating individual actions and can serve as powerful determinants of health behaviors (4). In sports contexts and health promotion settings, group norms can shape individuals’ attitudes and behaviors through mechanisms like conformity and peer pressure (5, 6). Team identification, grounded in social identity theory (7), refers to the psychological bond between individuals and their teams. Research suggests that strong team identification fosters adherence to team norms and enhances collective cohesion (8, 9). Individuals with high team identification are more likely to internalize team goals and align their behaviors with team values (10). Despite these findings, little is known about how group norms and team identification jointly influence health behaviors, particularly anti-doping practices, in collectivist cultural settings like China, where these social factors are especially pronounced. Understanding these mechanisms is crucial for developing culturally-appropriate health education programs.

The present study addresses this gap by investigating how group norms and team identification influence anti-doping behaviors among adolescent amateur athletes in Chinese sports schools. Specifically, we examine the direct effects of group norms on anti-doping behavior and the indirect effects through team identity. By revealing the pathways through which social dynamics influence health behaviors in this unique setting, this study aims to provide evidence-based guidance for developing culturally-adapted anti-doping health education and promotion programs. The findings have practical implications for health educators, coaches, sports administrators, and policymakers involved in youth health promotion. Moreover, this research contributes to advancing health education practice by illustrating how social factors can be strategically leveraged to enhance health promotion outcomes in organized sports settings, supporting collaborative efforts aligned with SDG 17 (Partnerships for the Goals) to promote adolescent health and well-being.

## 2 Literature review

### 2.1 The impact of group norms on anti-doping behavior

Group norms, defined by the collective values, beliefs, and behavioral standards within a group, play a crucial role in regulating individual actions and preserving group unity and stability (4). According to Kelman (11), the adherence to group norms by individuals progresses through three distinct phases: compliance, identification, and internalization. Studies have illustrated that group norms exert a significant influence on members’ judgments and attitudes, often through the pressure to conform (5), and they govern a range of behaviors, from deviant to prosocial (6).

Within the realm of sports, group norms impact athletes’ conduct through the perceived expectations of peers, coaches, and other relevant parties (12). Research indicates that athletes are more inclined to follow anti-doping regulations when group norms advocate for healthy sports practices. However, if group norms tolerate rule-breaking, this can result in the proliferation of deviant behavior (13). The rule-breaking actions of peers within the group can also subtly shape athletes’ perspectives. Erickson et al. (3) discovered that athletes’ emotional acceptance of doping significantly increased after learning that an acquaintance is using performance-enhancing drugs, highlighting that group norms influence not only external behavioral constraints but also internal attitude transformations through the process of norm internalization.

### 2.2 The impact of team identification on athletes’ behavior

Team identification, grounded in social identity theory, refers to the emotional and value-based affiliation an individual has with their team (7). This affiliation strengthens team cohesion and motivates members to act in concert with the team’s interests by integrating personal and team goals (8). Wakefield et al. (9) suggest that strong team identification enhances member well-being and encourages the active internalization of team values and objectives. In sports, team identification deepens athletes’ commitment to the team’s core ethos, leading to behaviors that are more congruent with team norms.

It is important to recognize that the influence of team identification is situationally contingent. In social identity theory, individuals adjust their behavior based on team identification only when their team identity is activated (14). Sports competitions are a salient context for athletes’ team identity, where the internal team dynamics and external fan support, coupled with financial incentives, all intensify the motivational impact of team identification on individual behavior (15). Dumas et al. (16) indicate that higher identification with certain groups leads to greater alignment of an individual’s behavior with those groups’ norms, which, while promoting prosocial conduct, may also reinforce the occurrence of negative behaviors.

### 2.3 Interaction between group norms and team identification

The interplay between group norms and team identification is substantiated by social identity theory. Rathbone et al. (17) suggest that group norms influence members’ affiliation with the team via social identification, which subsequently strengthens compliance with group norms. Within this dynamic, individual behavior is subject to both the direct effects of group norms and the moderating influence of team identification. Masson et al. (10) found that individuals are more likely to actively embody group norms when they perceive these norms positively through team identification. Reicher et al. (18) note that adherence to group norms is a means for individuals to assert their group membership, thereby reinforcing the emotional connection to the group during the exercise of social identity.

In sports, team identification often acts as a pivotal antecedent to group norms. Studies reveal that athletes with robust team identification are more compliant with group norms and more inclined to adjust their behavior to align with team objectives (19). Furthermore, athletes who foster strong trust with coaches or teammates are more likely to internalize group norms, treating them as personal behavioral standards (13). However, research on the combined effects of group norms and team identification on anti-doping behavior is sparse, especially within the context of collectivist cultures where in-depth research is lacking.

To encapsulate, group norms and team identification have been illustrated to significantly influence athletes’ behavior, but current research has mainly centered on Western cultural settings and professional athletes. There is a paucity of research on the anti-doping behavior of young athletes in amateur sports schools. Additionally, the interaction between group norms and team identification in collectivist cultures warrants further exploration. This study seeks to elucidate the relationship between group norms, team identification, and anti-doping behavior, aiming to fill the theoretical void in this area and to provide actionable insights for the development of anti-doping education strategies that are culturally sensitive to the Chinese context.

## 3 Research hypotheses

Building on prior literature and theoretical frameworks, this study investigates the relationship between group norms, team identification, and anti-doping behaviors among adolescent amateur athletes in China. Specifically, the study examines how group norms influence anti-doping behaviors directly and indirectly through team identification as a mediating variable. The following hypotheses are proposed:

### 3.1 Hypothesis 1: group norms and anti-doping behaviors

Group norms refer to the shared values, beliefs, and behavioral expectations that guide the actions of individuals within a group (4). Social influence theories suggest that individuals conform to group norms due to compliance, identification, or internalization (11). For adolescent athletes, the collectivist culture prevalent in Chinese sports training environments amplifies the influence of group norms, which act as implicit rules that regulate members’ attitudes and behaviors.

Prior studies have illustrated the significant impact of group norms on individuals’ decision-making processes, including risky behaviors such as smoking and substance abuse (6). In sports contexts, group norms have been shown to encourage pro-social behaviors, including adherence to anti-doping practices (12). Thus, this study posits:

*H1*: Group norms are positively associated with anti-doping behaviors among adolescent amateur athletes.

### 3.2 Hypothesis 2: group norms and team identification

Group norms are defined as the behavioral standards and expectations that are collectively accepted by team members (20). These norms can significantly affect the behavior of team members and the team’s sense of identity. Research has shown that group norms are one of the important antecedent variables in the formation of sports team identity.

The concept of team identity develops from the individual level to the collective level. The theoretical community generally defines team identity as the sense of belonging to the team identity or the perceived oneness with the team identity (21). Ellemers et al. (22) have provided detailed explanation of the concept of team identity from cognitive, affective, and evaluative dimensions. Team identity is when individuals regard the team as an object of belonging, recognize themselves as team members, and consider the team’s goals, interests, and norms as their own attributes.

Group norms have a significant impact on team effectiveness and team identity. Team norms, team cohesion, and team communication have significant impacts on team members’ satisfaction and their willingness to stay in the team (23). Group norms also influence team decision-making processes (24) and team motivation (46). Research on cultural differences indicates that collectivists emphasize conformity with the group and compliance with social group norms (25), suggesting that the impact of group norms on team identity may vary in different cultural contexts. Thus, Hypothesis 2 is proposed:

*H2*: Group norms are positively associated with team identification among adolescent amateur athletes.

### 3.3 Hypothesis 3: team identification and anti-doping behaviors

Team identification, grounded in social identity theory (7), signifies an individual’s emotional and cognitive attachment to their team. Athletes who possess a strong team identification are more inclined to internalize the team’s values and norms, thereby nurturing pro-social behaviors that are congruent with the team’s objectives (9).

Within the context of sports competitions, athletes’ team identities become particularly pronounced, amplifying the impact of team values and goals on individual actions. Extant literature underscores that robust team identification not only fortifies cohesion but also encourages adherence to ethical standards, including principles of fair play and anti-doping initiatives (8). Consequently, the third hypothesis is posited as follows:

*H3*: Team identification is positively associated with anti-doping behaviors among adolescent amateur athletes.

### 3.4 Hypothesis 4: team identification mediates the relationship between group norms and anti-doping behavior

The interplay between group norms and team identification has been a focal point in numerous behavioral studies. Social identity practice theory posits that individuals internalize group norms as an integral component of their social identity, employing these norms to navigate behavior and consolidate their sense of group belonging (17). In the domain of sports, team identification functions as a psychological mechanism through which group norms are translated into individual behaviors.

Within the collectivist framework of sports schools, adolescent athletes often illustrate a robust correlation between group norms and team identification. As the identification with their team intensifies, athletes become increasingly inclined to conform to group norms, thereby shaping their stance on anti-doping practices. Consequently, the fourth hypothesis is articulated as follows:

*H4*: Team identification plays a mediating role in the association between group norms and anti-doping behaviors.

## 4 Methodology

### 4.1 Questionnaire design

To enhance the credibility of the research findings, data were collected using a questionnaire based survey design. Given the distinctive institutional structure and cultural context of youth amateur sports schools in China, which differ substantially from training systems for youth amateur athletes in other countries, the questionnaire development process emphasized both cultural appropriateness and contextual relevance.

Accordingly, the questionnaire was developed through two complementary approaches. First, existing validated scales (26–29) were adapted to the Chinese youth sports school context. Specifically, The Group Norms and Team Identity Scale for Youth Amateur Athletes was revised on the basis of established measures, including the Social Identity Scale and the Group Norms Scale, with modifications made to ensure cultural and contextual suitability. Second, the Youth Amateur Athletes Anti-Doping Behavioral Characteristics Questionnaire was independently developed to capture behavior patterns that are specific to the training environment and competitive realities of Chinese youth amateur athletes.

#### 4.1.1 The group norms and team identity scale for youth amateur athletes

The Group Norms and Team Identification Scale for Youth Amateur Athletes was developed through a systematic process of adaptation and validation. In this study, group norms and team identification were operationalized as domain-specific constructs situated within the anti-doping context, with all items explicitly referring to doping-related norms, values, and behaviors. First, the original scales were translated and culturally adapted into Chinese with the assistance of a senior translator. To ensure linguistic equivalence and semantic clarity, follow-up interviews were conducted with coaches and athletes from the target population. Second, the scale items were refined through literature review, interviews, open-ended questionnaires, item categorization, and two rounds of expert consultation. Items deemed unsuitable were removed based on quantitative analyses, resulting in the final version of the questionnaire.

Internal consistency reliability was assessed using Cronbach’s alpha. The reliability coefficient for the group norms scale was 0.734, for the team identification scale was 0.727, and for the overall scale was 0.713, indicating acceptable internal consistency for all constructs. Although some subscales yielded Cronbach’s alpha values around 0.72, this level of internal consistency is consistent with current psychometric practices in athlete-focused and adolescent sport research. Previous validation studies of sport-specific psychological instruments have reported comparable reliability coefficients in the range of approximately 0.70–0.75 [e.g., (30, 31)]. Taken together, these findings suggest that the reliability levels observed in the present study are appropriate for context-specific psychological and behavioral measures in youth sport populations.

To further examine construct validity, confirmatory factor analysis was conducted to assess convergent validity. The group norms construct was specified as a three-dimensional structure, comprising intra-team norms, follower paths, and coalitions of interest. Intra-team norms refer to shared behavioral expectations and moral standards regarding doping within the team. Follower paths capture athletes’ tendencies to align their attitudes and behaviors with perceived majority views among teammates. Coalitions of interest reflect perceived collective priorities in which team success or performance goals may take precedence over anti-doping rules.

Team identification was conceptualized as a multidimensional construct reflecting athletes’ psychological attachment to their team, including identity process, identity influences, and identity crisis. Identity process represents the internalization of team-related values and ethical standards concerning doping. Identity influences capture external pressures and contextual factors shaping the strength and direction of team identification. Identity crisis reflects internal tension or ambivalence arising when personal beliefs about fairness or health conflict with perceived team expectations.

Following established criteria, standardized factor loadings exceeded 0.50 and were statistically significant, composite reliability values were greater than 0.70, and average variance extracted (AVE) values exceeded 0.50. For the group norms construct, all five items loading on Factor 1 (Intra-team Norms) showed factor loadings greater than 0.559, with an AVE of 0.526 and composite reliability of 0.841. Factor 2 (Follower Paths) demonstrated factor loadings above 0.588, an AVE of 0.513, and composite reliability of 0.799. Factor 3 (Coalitions of Interest) showed factor loadings above 0.643, with an AVE of 0.576 and composite reliability of 0.852. Similarly, within the team identification construct, all five items loading on Factor 1 (Identity Process) exceeded 0.568, with an AVE of 0.528 and composite reliability of 0.870. Factor 2 (Identity Influences) demonstrated factor loadings above 0.640, an AVE of 0.667, and composite reliability of 0.886. Factor 3 (Identity Crisis) showed factor loadings above 0.634, with an AVE of 0.645 and composite reliability of 0.813. These results indicate satisfactory convergent validity for both the group norms and team identification scales.

#### 4.1.2 The questionnaire on the characterization of anti-doping behavior of young amateur athletes

In this study, anti-doping behavioral characteristics were conceptualized as a multidimensional construct reflecting adolescents’ behavioral tendencies, proactive engagement, and resistance strategies in doping-related situations. Four dimensions were identified. Usage tendency refers to athletes’ subjective willingness to use doping substances or recommend them to teammates in performance-critical situations, capturing susceptibility to doping under competitive pressure. Information engagement reflects athletes’ proactive attention to and dissemination of anti-doping information within their teams, indicating active endorsement and promotion of anti-doping values. Opposition within the team describes athletes’ willingness to discourage, report, or intervene when teammates engage in doping behaviors, representing resistance to doping at the interpersonal and team level. Resolute resistance captures athletes’ firm adherence to anti-doping principles when facing explicit pressure or inducement, including refusal to use doping and prioritization of health and ethical standards over performance outcomes.

The Questionnaire on the Characterization of Anti-Doping Behavior of Young Amateur Athletes was developed independently using a categorical item design framework. Items were constructed to capture key behavioral responses across doping-related scenarios, including performance-critical decision-making, peer influence, ethical conflict, and proactive engagement in anti-doping actions. The questionnaire underwent two rounds of expert review to assess content relevance and conceptual clarity, resulting in a pilot version with satisfactory reliability and validity.

The pilot questionnaire was subsequently tested among youth amateur athletes in sports schools in Shenyang, and the same procedures were applied across two rounds of testing to further refine the instrument. Exploratory factor analysis was conducted using orthogonal rotation, with eigenvalues greater than 1 as the extraction criterion. The final scale consisted of 15 items loading on a single factor, consistent with the overall conceptualization of anti-doping behavioral characteristics. The scale demonstrated high internal consistency (Cronbach’s *α* = 0.899), with satisfactory sampling adequacy (KMO = 0.867, *p* < 0.001) and a cumulative explained variance of 86.942%, indicating good reliability and construct validity.

### 4.2 Subjects and sample

A convenience sampling approach was adopted in this study. First, provinces and cities were selected according to major geographic regions of China. Second, amateur sports schools were stratified within the selected provinces and cities. Finally, representative amateur sports schools were selected as the study sites.

To ensure the scientific rigor and reliability of the data collection process, all on-site surveys were administered by trained members of the research team. Prior to formal data collection, multiple preparatory meetings and standardized training sessions were conducted to familiarize investigators with the study objectives, core constructs, and questionnaire structure. During data collection, participants were informed of the voluntary and anonymous nature of the study, and written informed consent was obtained before questionnaire administration. Surveys were completed in supervised and quiet settings to minimize peer influence, and completed questionnaires were checked on site for missing responses or abnormal response patterns to enhance data quality.

The questionnaire survey was conducted using a combination of on-site surveys with paper questionnaires and online surveys with electronic versions of questionnaires. This study completed the survey of 15 cities, including Beijing (valid sample of 119), Yulin (29), Jinan (108), Kunming (127), Shanghai (266), Changchun (62), Changzhou (121), Fuyang (85), Zhaoqing (129), Taiyuan (228), Shijiazhuang (72), Nan’an (121), Xi’an (91), Wuhan (89), Shenyang (71), etc. The survey work of young amateur athletes in amateur sports schools, a total of 2,169 questionnaires were collected, and 1718 valid questionnaires were recovered. Participation in this study was entirely voluntary. Before completing the questionnaire, all participants provided informed consent after receiving a detailed explanation of the study’s objectives and purpose. They were explicitly informed that their data would be used solely for academic research (see Table 1).

**Table 1:** Anti-doping survey of students in amateur sports schools (*n* = 1718).

Serial number	District schools	Number of releases	Number of recoveries	Number of effectives	Number of invalidations
1	Beijing	142	142	119	23
2	Yulin	96	96	29	67
3	Jinan	145	145	108	37
4	Kunming	134	134	127	7
5	Shanghai	307	307	266	41
6	Changchun	97	97	62	35
7	Changzhou	150	150	121	29
8	Fuyang	113	113	85	28
9	Zhaoqing	171	171	129	42
10	Taiyuan	277	277	228	49
11	Shijiazhuang	109	109	72	37
12	Nan’an	126	126	121	5
13	Xi’an	102	102	91	11
14	Wuhan	100	100	89	11
15	Shenyang	100	100	71	29
Total		2,169	2,169	1718	451

The number of valid samples in the above 15 regions is 1718, with an average age of about 18 years old, 943 males and 775 females. From the distribution of specialties, track and field had the most members, 149, accounting for 8.6% of the total; among other sports, 111 were weightlifters, 106 were taekwondo players, 115 were boxers, and more than 50 other sports were included, such as basketball, wushu, sparring, fencing, cycling, badminton, rowing, and so on. In terms of sports grades, Grade 2 or below was the most popular, with 1,261, or 73.4%; Grade 1, 368, or 21.4%; and only 89, or 5.2%, were fitness generals.

Given the multi-site nature of the data collection, athletes in the present study were nested within teams and amateur sports schools. However, the analyses were conducted at the individual level to examine associations among perceived group norms, team identification, and anti-doping behaviors, which were the primary focus of this study.

## 5 Data analysis

After manual entry and proofreading of the collected data, SPSS 22.0 was used to perform statistical analyses such as common method bias test, test of difference in means and correlation analysis, and AMOS 22.0 was used to complete the modeling of structural equations and testing of mediating effects. Based on the scientific treatment of missing values and outliers, the questionnaire data were tested for normality, and the distribution of the variables was examined using the skewness and kurtosis methods. The results showed that the absolute values of skewness and kurtosis of all variables were less than 1, indicating that the distribution of the variables did not have obvious skewness and spikes, and could be considered as approximately obeying a normal distribution. Measurement reliability and validity tests were conducted on the questionnaire data, using Cronbach’s alpha coefficient, combined reliability (CR), average variance extracted (AVE), and factor analysis to detect the internal consistency, convergent validity, and discriminant validity of the variables. It was found that the Cronbach’s alpha coefficient and CR value of all variables were greater than 0.7, indicating that the variables had high internal consistency; the AVE value of all variables was greater than 0.5, indicating that the variables had high convergent validity; the square root of the AVE of all variables was greater than the correlation coefficients between the variable and other variables, indicating that the variables had high discriminant validity. The square root of AVE for all variables is greater than the correlation coefficient between the variable and other variables, indicating that the variables have high discriminant validity. Therefore, the formal questionnaire of this study has good reliability and validity and can be analyzed by structural equation modeling.

### 5.1 Factor analysis

Exploratory factor analysis showed that: the sports team identity dimension mainly includes three factors: team identity influencing factors, identity crisis and identity process; the group norms dimension mainly includes three factors: herd path, in-group norms and group alliance of interest; and the dimension of anti-doping behavioral characteristics of youth amateur athletes includes four factors: use tendency, information propaganda, intra-team opposition and resolute boycott (see Tables 2–4).

**Table 2:** Factor loadings and item descriptions for the group norms.

Construct	Subject	Joint explanation of variance	KMO	*p*
Intra-group norms	E1, E7, E9, E12, E14	88.972%	0.867	0.000
Herd path	E2, E5, E8, E10, E15	72.252%	0.852	0.000
Coalition of group interests	E3, E4, E6, E11, E13	74.252%	0.832	0.000

**Table 3 tab3:** Factor loadings and item descriptions for the team identification.

Construct	Subject	Joint explanation of variance	KMO	*p*
Team identity process	E18, E21, E24, E27, E30	77.350%	0.843	0.000
Team identity influences	E17, E20, E23, E26, E29	75.422%	0.840	0.000
Team identity crisis	E16, E19, E22, E25, E28	76.232%	0.832	0.000

**Table 4:** Factor loadings and item descriptions for anti-doping behavioral.

Construct	Subject	Joint explanation of variance	KMO	*p*
Usage tendency	F1, F3, F5, F9	83.737	0.710	0.000
Information propaganda	F6, F7, F8, F11	79.270	0.756	0.000
Opposition within the team	F2, F12, F13, F14	78.133	0.782	0.000
Resolute resistance	F4, F10, F15	76.971	0.797	0.000

On this basis, this study attempts to develop a study on the influence of group norms on behavior based on the mediation of team identity from the perspective of adolescent amateur athletes, using structural equation modeling to develop, estimate, and test causal models.

### 5.2 Presumptive structural modeling and its testing

**Figure 1:**
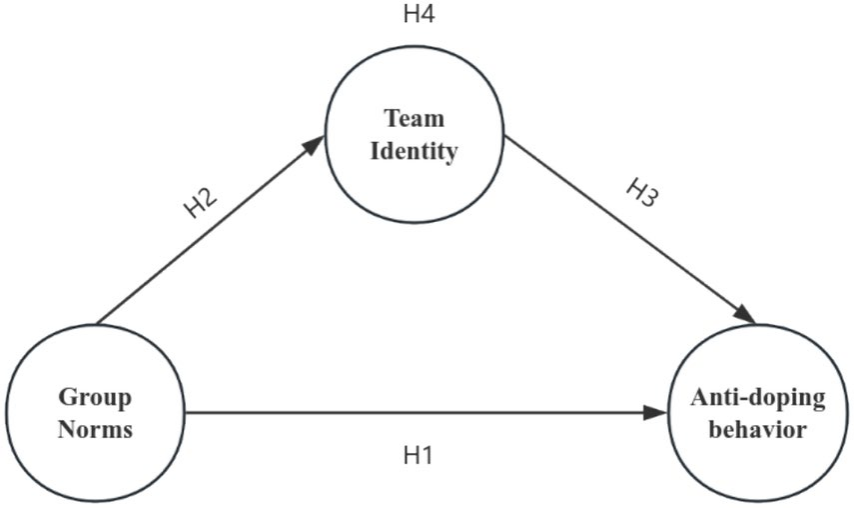
Theoretical presupposition model of this study.

Based on the hypotheses proposed in this study, a prespecified structural equation model of “the influence of group norms based on team identity mediation on the anti-doping behavior of young amateur athletes” was attempted to be constructed, as shown in Figure 1.

The inspection of the structural model begins with a test for violation of model identification. The basic fitness indexes of the model meet the standard requirements of the test and evaluation program. On the basis of the above, the fitness test is carried out on the preset structural equation model. The two types of fit indexes, absolute fit index and value-added fitness index, meet the requirements of model construction, indicating that the preset structural equation model constructed in this study fits well with the actual data, see Table 5.

**Table 5:** Overall model fit indices of the structural equation model.

Fitting index	Absolute fit index			Value-added fit index					
	*x*^2^/df	GFI	RMSEA	AGFI	NFI	CFI	IFI	RFI	TLI
Ideal value	1–3	>0.9	<0.08	>0.9	>0.9	>0.9	>0.9	>0.9	>0.9
Indicator value	3.899	0.966	0.068	0.961	0.961	0.966	0.966	0.920	0.929

Usually, *x*^2^/df <3, the model is better; when the sample is larger, the criterion of reasonableness can be relaxed to 5, and the model is acceptable if the measured data basically fit the model.

### 5.3 Structural equation modeling

**Figure 2:**
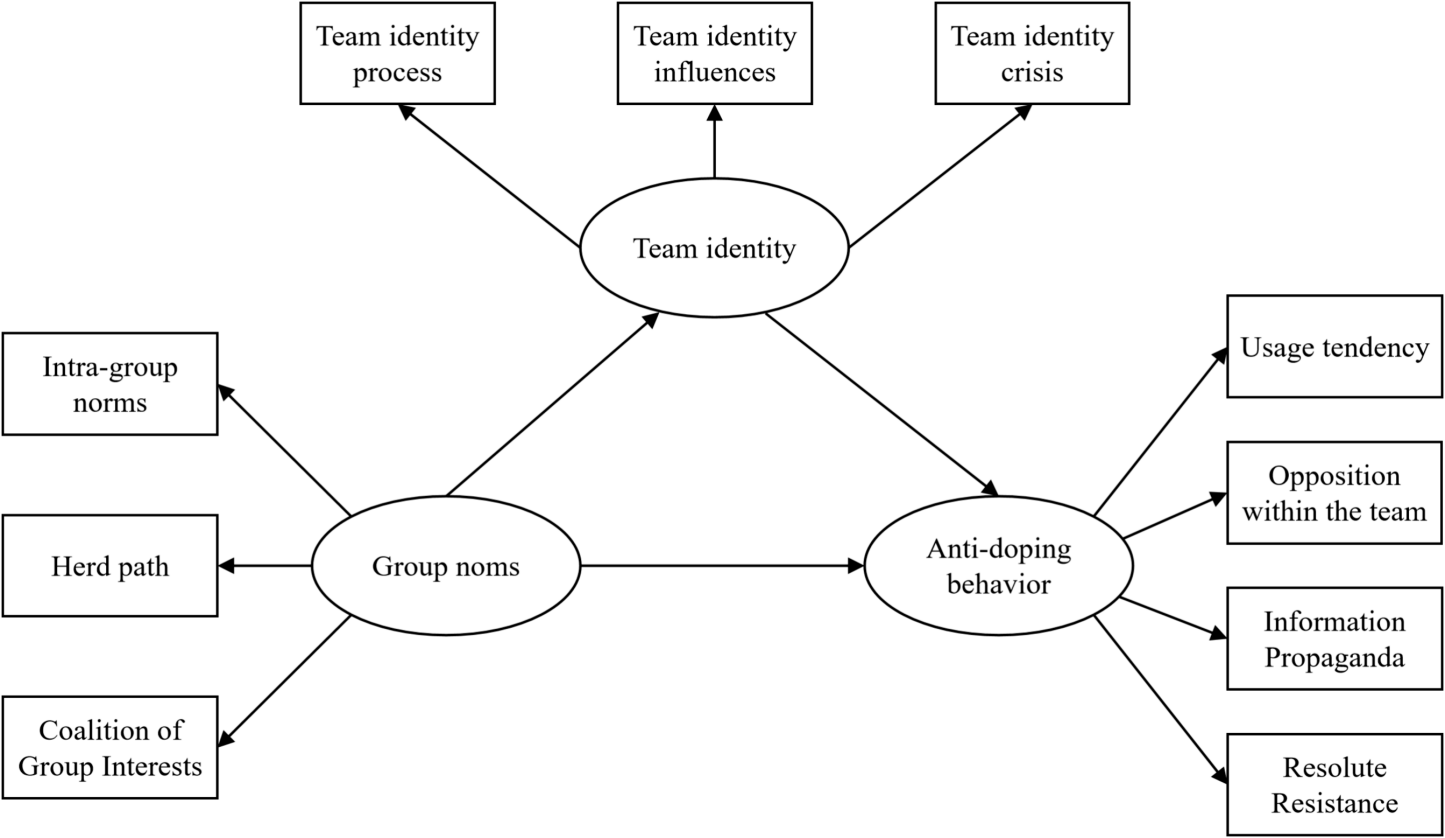
Preset structural equation model.

According to the structural equation model constructed in this study (see Figure 2), the results indicate that group norms are positively and significantly associated with anti-doping behaviors among youth amateur athletes. The standardized regression coefficient between group norms and anti-doping behavior was 0.54, supporting Hypothesis 1 (see Table 5). This finding suggests that higher levels of identification with group norms are associated with stronger engagement in anti-doping behaviors among youth amateur athletes.

**Figure 3:**
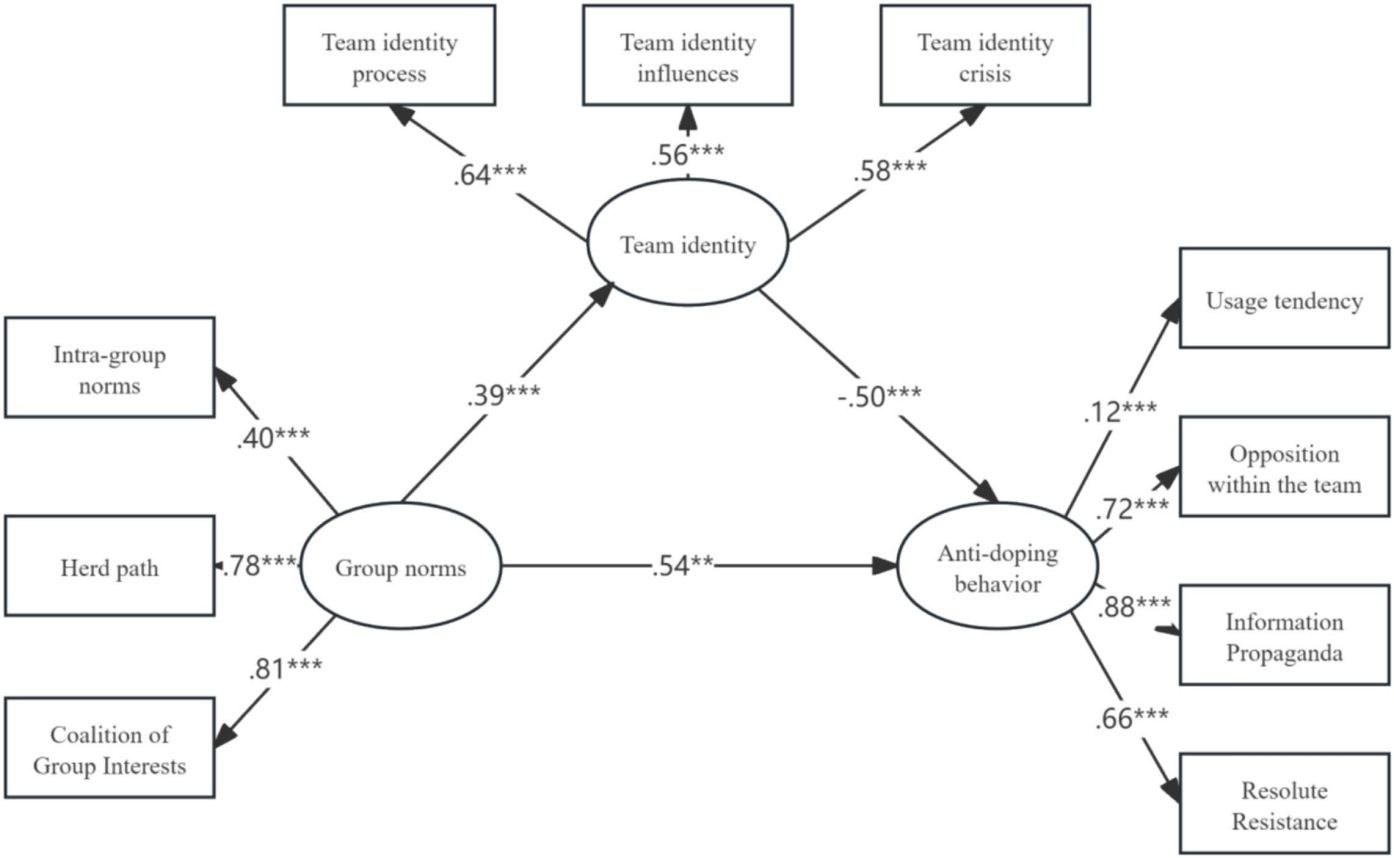
SEM of group norms, team identification, and anti-doping behaviors.

Mediation analysis further showed that team identification plays a partial mediating role in the association between group norms and anti-doping behaviors. In the confirmatory factor analysis and structural equation modeling, no item parceling was applied, and all latent constructs were specified as first-order factors indicated by their corresponding observed subscale items (see Figure 3, Table 6).

**Table 6:** Path coefficients and hypothesis testing results of SEM.

Hypothesis	*p*	Standardized path coefficient estimate	Rejection/acceptance
Team identity ← group norms	***	0.39	Acceptance
Anti-doping behavior ← group norms	***	0.54	Acceptance
Anti-doping behavior ← team identity	**	−0.50	Acceptance

^*^indicates significance at the 0.05 level, ^**^indicates significance at the 0.01 level, and ^***^indicates significance at the 0.001 level.

Following contemporary mediation analysis practices, the mediating role of team identification was evaluated primarily based on bias-corrected bootstrapped indirect effects. For completeness, the traditional three-step framework proposed by Baron and Kenny (32) was used as a conceptual reference. The standardized path estimates showed that group norms were positively associated with team identification (standardized coefficient = 0.39), providing support for Hypothesis 3. In addition, team identification was negatively associated with anti-doping behaviors (standardized coefficient = −0.50), providing support for Hypothesis 4 and indicating a partial mediation pattern (see Table 3).

To formally assess the indirect effect, a bias-corrected bootstrapping procedure with 2,000 resamples and a 95% confidence interval was conducted following Preacher and Hayes (33). The indirect effect of team identification was −0.194, with a 95% confidence interval of [−0.312, −0.117], which did not include zero, indicating a statistically significant mediation effect. The direct effect of group norms on anti-doping behavior was 0.542, and the indirect effect accounted for 35.8% of the total effect. Taken together, these results indicate that group norms are associated with anti-doping behaviors both directly and indirectly through team identification, providing support for Hypothesis 2.

### 5.4 Analysis of results

The results of exploratory factor analysis showed that all three variables (group norms, team identity, and anti-doping behavior) in this study could be extracted as specific factors separately, and their respective factor loadings were all greater than 0.5, indicating that the measurement items of each variable had high internal consistency; the variance explained by each factor was greater than 70%, indicating that the factors had high convergent validity; and the correlation coefficients between each factor were all less than 0.8, indicating that the factors have high discriminant validity. Therefore, the results of the exploratory factor analysis of this study support the factor structure and measurement validity of this study.

The results of the structural equation modeling analysis showed that the model in this study was well fitted, and all the fit indicators reached the desired level. The path coefficients and significance levels of the model indicate that group norms have a positive direct effect on anti-doping behavior (*β* = 0.54, *p* < 0.001); team identification has a negative direct effect on anti-doping behavior (*β* = −0.50, *p* < 0.001) and a negative mediating effect (*β* = −0.35, *p* < 0.001), between group norms and anti-doping behavior played a partially mediating role. Therefore, the results of the structural equation modeling analysis in this study supported the three hypotheses and the fit and validity of the model.

## 6 Discussion

This study examined the association between group norms and anti-doping behaviors among adolescent amateur athletes in sports school settings, with team identification serving as a mediating variable. From a health promotion perspective, elucidating how social and identity-based processes shape health-related behaviors is essential for designing evidence-based anti-doping education strategies that are sensitive to group dynamics in organized sport contexts. The findings revealed both opportunities and challenges for health education practice. Group norms illustrated a strong positive association with anti-doping behaviors (standardized regression coefficient = 0.54), indicating that social normative environments are closely linked to health-related behavioral patterns in adolescent athletes. Notably, and contrary to the initial hypothesis, team identification exhibited a significant negative mediating effect (indirect effect = −0.194, accounting for 35.8% of the total effect), representing the most innovative contribution of this study and highlighting a counterintuitive social dynamic underlying anti-doping behaviors. Nevertheless, this finding suggests that health promotion interventions targeting adolescent doping behaviors should move beyond the assumption that stronger team cohesion and identification are inherently protective, and instead attend to how team identities are constructed, which values they prioritize, and under what conditions they may inadvertently weaken alignment with health-promoting norms in competitive sport environments.

### 6.1 Group norms as a strong determinant of anti-doping behaviors

The positive association between group norms and anti-doping behaviors underscores the theoretical importance of norm-based mechanisms in shaping health-related behaviors among youth amateur athletes. Group norms, developed through shared training experiences and collective interactions, function as regulatory forces that guide individual attitudes and behavioral choices. Consistent with classic findings on social conformity, normative pressure can meaningfully influence decision-making processes, including judgments involving ethical considerations.

Previous research on doping behaviors has primarily emphasized individual-level psychological determinants, particularly within the Theory of Planned Behavior framework (34–36). The present study extends this line of research by situating subjective norms within a broader group-based social environment. By focusing on group norms embedded in team contexts, the findings illustrate that normative influences operate not only as perceived expectations but also as structural features of the social environment that shape behavior through both external regulation and internalized identification processes (13, 47).

The magnitude of the observed association (*β* = 0.54) highlights the relevance of group norms as a central mechanism linking social context to anti-doping behaviors. Among adolescent athletes, normative influences arising from training groups, including conformity pressures and shared goal orientations, may affect multiple cognitive and behavioral processes, such as attitudes toward doping, willingness to oppose doping within the team, dissemination of anti-doping information, and peer monitoring. From a health education perspective, these results suggest that interventions targeting group-level normative structures may be particularly effective, providing multiple entry points for promoting clean sport through peer modeling, team culture development, and norm-consistent organizational practices.

### 6.2 The role of group norms in strengthening team identification

This study confirmed that group norms significantly influence team identification (*β* = 0.39, *p* < 0.001), illustrating that normative environments serve as important antecedents shaping the strength of athletes’ psychological bonds with their teams. Through exploratory factor analysis, we identified three key dimensions of group norms, namely in group norms, followership paths, and coalitions of interest, each exerting a distinct influence on the formation of team identification. The formation of group norms is not a static condition but a gradual social process, developing through stages such as social cues, imitation, conformity, internalization, and identification. In this process, conformity plays a particularly important role by guiding young athletes to align their attitudes and behaviors with perceived team expectations. Notably, coalitions of interest exerted the strongest influence on team identification, followed closely by followership paths, while in group norms illustrated a comparatively weaker but still significant effect. These dimensions reflect different aspects of team attributes, behaviors, and attitudes, with in group norms capturing perceived stability and legitimacy, followership paths highlighting comparative group distinctiveness, and coalitions of interest representing relational and goal based team composition. This pattern has important implications for understanding the internal social structures of sports teams, where shared values, interpersonal relationships, and collective goals are closely intertwined. Consistent with social identity theory, team attributes such as perceived status, stability, continuity, and legitimacy play a central role in shaping team identification, particularly in structured youth sports team contexts.

### 6.3 Team identity undermines health-promoting norms: explaining the negative mediation effect

However, contrary to Hypothesis 3, team identification exhibited a significant negative mediating effect between group norms and anti-doping behaviors (indirect effect = −0.194, accounting for 35.8% of the total effect). This finding indicates that stronger alignment with the team does not necessarily translate into stronger adherence to anti-doping norms, suggesting that the role of team identification in health-related behavior regulation is more conditional and context dependent than initially assumed.

To understand this pattern, it is important to recognize that team identification functions primarily as a mechanism that intensifies alignment with dominant group values rather than as an inherently health-promoting force. Social identity theory suggests that identification strengthens conformity to group norms and shared goals, thereby amplifying collective orientations and coordinated action (7, 37). As a result, the behavioral consequences of team identification depend critically on the content of the values embedded within the team identity itself (38). When health protection and ethical conduct are central to the team identity, stronger identification may reinforce anti-doping behaviors. When these values are secondary to other priorities, the same identification process may produce the opposite effect.

This value dependent mechanism becomes particularly salient in environments where performance outcomes dominate the definition of success. Research on motivational climates distinguishes performance oriented contexts, which emphasize social comparison, evaluation, and competitive results, from mastery oriented contexts that prioritize skill development, learning, and long term well-being (39, 40). In performance oriented climates, strong team identification may heighten pressure to prioritize collective achievement and external recognition, even when doing so conflicts with health-promoting norms. Empirical studies in sport psychology suggest that such climates are more strongly associated with risk taking and ethically questionable behaviors, whereas mastery oriented climates are more likely to support moral functioning and health oriented decision making (41, 42).

For adolescent athletes, this dynamic is further intensified by developmental characteristics associated with identity formation. Adolescents are particularly sensitive to peer approval and group belonging, making them more likely to internalize team goals as personal goals and to equate loyalty with compliance. Within sports teams, where training is highly interdependent and individual behavior is closely tied to collective performance outcomes, resisting dominant team practices can carry substantial social costs. Consequently, strong team identification under performance oriented conditions may discourage questioning of team norms, even when athletes are aware of anti-doping principles.

Over time, this process may facilitate group level moral disengagement, whereby behaviors that conflict with broader ethical standards are cognitively justified in the name of collective success. Moral disengagement mechanisms such as diffusion of responsibility or moral justification allow individuals to reconcile participation in questionable practices with their self-concept as moral actors (43, 44). In sport settings, such group based moral disengagement has been shown to weaken the link between moral awareness and actual behavior, increasing vulnerability to norm violating actions including doping related behaviors (45).

Within Chinese youth amateur sports schools, this sequence of mechanisms may be particularly visible due to structured training systems, hierarchical coaching relationships, and strong collective performance orientations. However, these contextual features should be understood as intensifying conditions rather than unique causes. The negative mediating role of team identification observed in this study reflects a more general social psychological process that can emerge in high performance team environments across cultural contexts when strong identification aligns with performance priorities that conflict with health-promoting values.

Taken together, the findings indicate that team identification operates as a conditional social mechanism in adolescent sports teams. Its influence on anti-doping behaviors depends on how team success is defined and which values are embedded within the collective identity. By clarifying the conditions under which team identification undermines health-promoting norms, this study provides a theoretically integrated explanation for the observed negative mediation effect.

### 6.4 Implications for health education and anti-doping intervention design

The findings have important implications for health education and promotion practice in sports school settings, particularly given that strong team identification may unintentionally undermine anti-doping behaviors when team values are misaligned with health protection goals. The dual influence of group norms and team identification identified in this study suggests that effective anti-doping interventions must move beyond single level strategies and adopt integrated approaches that address both normative and identity based processes. While the establishment of clear anti-doping norms at the organizational level remains essential, such efforts may be insufficient if informal interest based coalitions within teams operate according to competing performance oriented values.

Accordingly, health educators and sports administrators should avoid assuming that strengthening team cohesion and identity is inherently beneficial. Instead, intervention efforts should focus on shaping team identities that explicitly incorporate health protection, ethical conduct, and long term athlete development as core values. This may involve embedding anti-doping principles into team building activities, aligning team goals and success criteria with health oriented outcomes, and actively engaging influential subgroups within teams to ensure that collective interests support clean sport practices. From a policy perspective, integrating anti-doping education into the core mission and daily culture of sports schools, rather than treating it solely as a compliance requirement, may help create supportive environments in which both group norms and team identities consistently promote anti-doping behaviors.

## 7 Limitations and future research recommendations

Several limitations of this study should be acknowledged. First, although the sample consisted of adolescent amateur athletes nested within teams and sports schools, the analyses were conducted at the individual level and did not explicitly model potential clustering effects. As a result, unobserved team- or school-level influences may have contributed to shared variance in the measured variables. Future research could address this issue by employing multilevel modeling or multilevel structural equation modeling to more clearly disentangle individual- and group-level processes.

Second, the sample focused on youth amateur sports school athletes from selected regions in China, which may limit the generalizability of the findings to other sport systems or cultural contexts. Expanding sample diversity across regions, sports, and cultural backgrounds would help to enhance external validity.

Third, the cross-sectional design precludes strong causal inferences, and the observed associations and mediation patterns should therefore be interpreted as statistical relationships rather than causal mechanisms. In addition, responses may have been influenced by social desirability bias given the sensitive nature of doping-related topics.

Fourth, although the measurement of group norms and team identification was grounded in established theoretical frameworks, these complex constructs may not have been fully captured across all possible dimensions. Future studies may benefit from integrating qualitative approaches to gain deeper insight into how social dynamics are experienced and negotiated within sport teams.

Finally, future research would benefit from longitudinal designs to better understand how group norms and team identification evolve over time and shape behavioral trajectories. Experimental or quasi-experimental studies testing norm-based and identity-based interventions would also provide valuable evidence for translating these findings into effective health promotion and anti-doping education practices for adolescent athletes.

## 8 Conclusion

This study reveals the mechanisms by which group norms and team identification influence anti-doping behaviors among adolescent amateur athletes in Chinese sports schools, providing new evidence for health education practice in collectivist sports settings. The findings indicate that group norms are powerful determinants of health behaviors, offering clear opportunities for norm-based health promotion interventions. However, the negative mediating effect of team identification introduces important complexity, revealing that social influence in sports settings operates through multiple, sometimes conflicting pathways. Even as sports schools work to establish anti-doping norms through policy and education, interest-based coalitions within teams may create pressures that complicate these efforts. This underscores the need for comprehensive, integrated health education approaches that simultaneously address normative environments and team identity processes, ensuring that as team bonds strengthen, they are anchored in health-promoting rather than performance-compromising values. By understanding and strategically leveraging these social dynamics, health educators, coaches, and administrators can develop more effective interventions to promote anti-doping behaviors and contribute to the health and well-being of adolescent athletes. These efforts support the achievement of Sustainable Development Goals related to health, education, and partnerships, advancing both individual athlete welfare and the integrity of youth sport.

## Data Availability

The raw data supporting the conclusions of this article will be made available by the authors, without undue reservation.
