# Supplementary material for: Promoting anti-doping behaviors through group norms: understanding the role of team identity among adolescent athletes
Source: Front Public Health. 2026 Feb 24;14:1788343. doi: 10.3389/fpubh.2026.1788343 (PMC12971910; doi:10.3389/fpubh.2026.1788343)
Supplement: Supplementary file 1 [file Data_Sheet_1.pdf]

Supplementary information

Additional file 1 of Group Norms and Team Identity Scale for Youth Amateur Athletes

Group Norms and Team Identity Scale for Youth Amateur Athletes

Dear student:

Please read carefully and reflect on whether the statements below align with your situation. Use numbers "1" to "7" to indicate the degree of agreement, where "1" means "Strongly Disagree," "7" means "Strongly Agree," and "2" to "6" represent intermediate degrees increasing progressively. Please write the number that corresponds to your level of agreement in the parentheses () before each question number. Thank you for your support and cooperation!

Example:

(1) You took this job because you have a strong interest in it.

1 2 3 4 5 6 7

If you feel "Strongly Disagree," please fill in "1" in the parentheses.

Strongly Disagree ←——→ Strongly Agree

( )E1 Even if doping harms health, we must comply with the coach's requirements for the sake of competition results. 1 2 3 4 5 6 7

( )E2 If everyone else is using doping and I am not, I would feel foolish.

1 2 3 4 5 6 7

( )E3 To ensure our team's victory, I would introduce the best doping methods to my teammates.

1 2 3 4 5 6 7

( )E4 I feel very pleased if I achieve excellent results in competitions without being drug-tested.

1 2 3 4 5 6 7

( )E5 If doping guarantees success and non-use leads to failure, I believe most people would take the risk.

1 2 3 4 5 6 7

( )E6 Coaches care most about performance results and are willing to try any method to improve them.

1 2 3 4 5 6 7

( )E7 Some people say doping violates sports ethics.

1 2 3 4 5 6 7

( )E8 If all teammates are using doping and I refuse, I fear being excluded.

1 2 3 4 5 6 7

( )E9 If I use doping, I will consult the coach for advice.

1 2 3 4 5 6 7

( )E10 Many peers believe that without doping, it's impossible to achieve good results—better to take a chance.

1 2 3 4 5 6 7

( )E11 If national honor is not involved, governments won't invest significant effort in anti-doping.

1 2 3 4 5 6 7

( )E12 If I use doping, I will not seek the coach's advice.

1 2 3 4 5 6 7

( )E13Leaders may sympathize with dopers caught but have no choice but to penalize them.

1 2 3 4 5 6 7

( )E14 If the coach asks me to dope, I will refuse.

1 2 3 4 5 6 7

( )E15If others use doping and I don't, I consider it smart.

1 2 3 4 5 6 7

( )E16 I know the consequences of doping.

1 2 3 4 5 6 7

( )E17 To achieve outstanding results, I sometimes disregard methods, including doping.

1 2 3 4 5 6 7

( )E18 I genuinely accept the ban on doping.

	1 2 3 4 5 6 7
( )E19 I am willing to join the anti-doping movement.	
	1 2 3 4 5 6 7
( )E20 I pin all hopes on doping for competition outcomes.	
	1 2 3 4 5 6 7
( )E21 Athletes who refuse doping and are expelled are acting foolishly.	
	1 2 3 4 5 6 7
( )E22 Doping violates the principle of "fair competition" and is unfair.	
	1 2 3 4 5 6 7
( )E23 Many athletes feel pressured to dope due to excessive societal expectations.	
	1 2 3 4 5 6 7
( )E24 Many doping behaviors are worthy of sympathy.	
	1 2 3 4 5 6 7
( )E25 If doping is detected, a lifetime ban and fines are too harsh—canceling results should suffice.	
	1 2 3 4 5 6 7
( )E26 Punishing only athletes for doping is unfair—coaches should also be held accountable.	
	1 2 3 4 5 6 7
( )E27 Coaches openly encouraging doping are selfish and shameful.	
	1 2 3 4 5 6 7
( )E28 I generally understand the harm of doping.	
	1 2 3 4 5 6 7
( )E29 Some claim doping is a deceptive way to gain honor.	
	1 2 3 4 5 6 7
( )E30 Athletes who refuse doping and are expelled are not worthy of pity.	
	1 2 3 4 5 6 7

Additional file 2 of The Questionnaire on the Characterization of Anti-Doping Behavior of Young Amateur Athletes

The Questionnaire on the Characterization of Anti-Doping Behavior of Young Amateur Athletes

Dear Student:

This questionnaire aims to understand the behavioral tendencies you may exhibit during training and competitions. Please read the following questions carefully and use numbers "1" to "7" to indicate the degree of alignment with your actual situation ("1" = "Strongly Disagree," "7" = "Strongly Agree"). Write the corresponding number in the parentheses before each question number.

Strongly Disagree ←→ Strongly Agree

( )F1 I would use doping during critical moments of a match.	1 2 3 4 5 6 7
( )F2 If I discover a teammate using doping, I will strongly discourage them.	1 2 3 4 5 6 7
( )F3 If certain doping methods cannot be detected by authorities, I would use them.	1 2 3 4 5 6 7
( )F4 I would prioritize integrity over victory and refuse to use doping.	1 2 3 4 5 6 7
( )F5 To secure team victory, I would recommend the most effective doping methods to my teammates.	1 2 3 4 5 6 7
( )F6 I regularly follow anti-doping information.	1 2 3 4 5 6 7
( )F7 If new anti-doping guidelines are released, I will promptly inform my coach and teammates.	1 2 3 4 5 6 7

( )F8 I frequently advise teammates about the dangers of doping.

1 2 3 4 5 6 7

( )F9 If team victory requires it, I would provide teammates with the most effective doping methods.

1 2 3 4 5 6 7

( )F10 If doping helped me achieve success but caused guilt, I would voluntarily disclose it to the tournament organizers.

1 2 3 4 5 6 7

( )F11 I would explain the health risks of doping to my teammates.

1 2 3 4 5 6 7

( )F12 I would explain the health risks of doping to my teammates.

1 2 3 4 5 6 7

( )F13 If I suspect a teammate is using doping, I will report them.

1 2 3 4 5 6 7

( )F14 If a teammate's success is linked to doping, I will expose their actions.

1 2 3 4 5 6 7

( )F15 If my coach mandates doping for health reasons, I will withdraw from the team.

1 2 3 4 5 6 7
